# Patients with Bicuspid Aortopathy and Aortic Dilatation

**DOI:** 10.3390/jcm11206002

**Published:** 2022-10-11

**Authors:** Francesco Nappi, Omar Giacinto, Mario Lusini, Marialuisa Garo, Claudio Caponio, Antonio Nenna, Pierluigi Nappi, Juliette Rousseau, Cristiano Spadaccio, Massimo Chello

**Affiliations:** 1 Department of Cardiac Surgery, Centre Cardiologique du Nord, 93200 Saint-Denis, France; 2 Department of Cardiovascular Surgery, Università Campus Bio-Medico di Roma, 00128 Rome, Italy; 3 Department of Clinical and Experimental Medicine, University of Messina, 98122 Messina, Italy; 4 Department of Cardiac Surgery, Massachusetts General Hospital & Harvard Medical School, Boston, MA 02115, USA

**Keywords:** bicuspid aortic valve, aortopathy, classification, diagnosis, treatment

## Abstract

(1) Background: Bicuspid aortic valve (BAV) is the most frequent congenital cardiac disease. Alteration of ascending aorta diameter is a consequence of shear stress alterations due to haemodynamic abnormalities developed from inadequate valve cusp coaptation. (2) Objective: This narrative review aims to discuss anatomical, pathophysiological, genetical, ultrasound, and radiological aspects of BAV disease, focusing on BAV classification related to imaging patterns and flux models involved in the onset and developing vessel dilatation. (3) Methods: A comprehensive search strategy was implemented in PubMed from January to May 2022. English language articles were selected independently by two authors and screened according to the following criteria. (4) Key Contents and Findings: Ultrasound scan is the primary step in the diagnostic flowchart identifying structural and doppler patterns of the valve. Computed tomography determines aortic vessel dimensions according to the anatomo-pathology of the valve. Magnetic resonance identifies hemodynamic alterations. New classifications and surgical indications derive from these diagnostic features. Currently, indications correlate morphological results, dissection risk factors, and genetic alterations. Surgical options vary from aortic valve and aortic vessel substitution to aortic valve repair according to the morphology of the valve. In selected patients, transcatheter aortic valve replacement has an even more impact on the treatment choice. (5) Conclusions: Different imaging approaches are an essential part of BAV diagnosis. Morphological classifications influence the surgical outcome.

## 1. Introduction

Bicuspid aortic valve (BAV) is the most frequent congenital cardiac pathology; has a prevalence of 1–2% [1], a high incidence of adverse outcomes, especially aortic stenosis (AS) and aortic regurgitation (MR) [2]; and is at least three times more common in males than females [3]. 

Bicuspid aortopathy, reported in 50% of BAV patients, consists of the aorta enlargement starting from the aortic root and involving the aortic arch and depends on blood flux turbulences characterized by power vectors directed against the aortic toot and the convexity of the vessel [4,5,6,7]. Recently, micro-RNA (miRNA) has been studied regarding post-transcriptional regulation of genes in aortopathy manifestation. [8,9]. This paper aims to discuss the current knowledge about anatomical, pathophysiological, genetical, ultrasound, and radiological aspects of BAV disease, focusing on BAV classification related to imaging patterns and flux models involved in the onset of aortic dilatation and its developed process. We present the following article in accordance with the narrative review reporting checklist.

## 2. Methods

This narrative review was carried out from January 2022 to May 2022. The following search strategy was implemented on PubMed: (BAV OR bicuspid aortopathy OR bicuspid aortic valve) AND (ultrasound OR computed tomography OR magnetic resonance OR US OR CT or MR). Published articles were evaluated from database inception up to search date. Only articles in the English language were included. Details are reported in Table 1.

## 3. Genetics and Molecular Biology 

Estimating mutation genes and their inheritance patterns is challenging [7] because locus 9q34.3 alteration causes mutations in regulators NOTCH1 with secondary pathological aortic valve development [10,11]; gene damages on 18q, 5q, and 13q induces BAV [12]; and finally, damages to the smooth muscle alfa actine (ACTA 2) gene produce BAV and aortic aneurysms [13].

There is a tight linkage between BAV expression and other congenital pathologies such as the coarctation of the aorta. Concerning BAV phenotype, Shone’s syndrome with a left-sided lesion that can cause inflow and outflow obstruction, Turner’s syndrome with aortic coarctation, and William’s syndrome involving supravalvular stenosis may be observed. Moreover, ventricular septal defect, atrial septal defect, patent ductus arteriosus, and coronary vessels, which may mainly involve single coronary and reversal coronary dominance, have been reported [14,15,16].

Micro-RNAs (MiRNAs) need to be considered in biochemical and molecular changes in BAV and aorthopathy (Table 2). MiRNAs are small, single-stranded, noncoding RNA molecules that determine the post-transcriptional regulation of gene expression. The effects of miRNAs are the result of base pairing with complementary sequences within mRNA molecules that are silenced by cleavage of the mRNA strand, destabilization of the mRNA by shortening its tail, and less efficient translation into proteins by ribosomes [17]. MiRNA expression profiling studies show that the expression levels of certain miRNAs change in diseased human hearts, suggesting their involvement in cardiomyopathies. MiR-712 is a potential predictor of atherosclerosis, has blood flow-dependent expression, and miR-712 is also upregulated in endothelial cells exposed to naturally occurring d-flow in the greater curvature of the aortic arch [18]. Several studies have investigated the cooperation of miRNA, metalloproteinases (MMP), and tissue inhibitor of matrix metalloproteinases (TIMP) in aorthopathy secondary to morphological alteration of the aortic valve. miRNAs related to dilation of the thoracic aorta (TA) are upregulated in transcriptional and epigenetic ways: different levels of MMP-2, MMP-9, TIMP-1, and TIMP-9 were observed [19]. A high level of MMP-2 and increased levels of miR-17 and miRNAs with the same genetic features as miR-17 were found in a comparative study involving patients with mild and severe aorta dilation, with a decreased level of TIMP -1, TIMP-2, and TIMP-3, thus hypothesizing a continuous development of TA influenced by BAV [20]. A recent study showed a relationship between miR-133a and TIMP-1 and TIMP-2 without reporting a statistically significant association between miR-143 and MMP-2 [21]. 

Plasma exosomal miR-423-5p regulates TGF-β signaling by targeting “similar mothers against decapentaplegic Drosophila gene” 2 (SMAD2), exerting functions in the initiation and development of BAV disease and its complication, bicuspid aortopathy [22,23]. Circulating miRNAs may reflect remodeling processes in the proximal aorta in patients with bicuspid aortopathy, and a recent study found a significant association between miRNA expression in peripheral blood and aortic tissue, as levels of miR-21, miR-133a, miR-143, and miR-145 were associated with dilated aorta [24].

Since abnormalities in vascular smooth muscle cells (VSMCs) may influence the development of TA dilation, primarily when contractile function converts to secretory function, this molecular situation causes cell apoptosis, in which the role of miRNA regulation may play a crucial role. Specifically, the convex part of ascending thoracic aorta (ATA) in BAV has increased miR-146-5p and miR-21-5p and reduced miR-133a-3p levels [25]; miR-424-3p and miR-3688-3p are downregulated in Hippo, ErbB, and TGF-beta signalling pathways, an epiphenomenon of cell proliferation and apoptosis [26]; and, finally, endothelial cells may have alterations due to abnormal flux patterns and genetic factors. This last alteration results in a less resistant vessel wall and can start a process of aortic dilation. Moreover, miR-494 is associated with platelet endothelial cell adhesion molecule (PECAM) and microparticles derived from endothelial cells [26], and the decreased expression of the miR-200 group can determine the involvement of the miR-200 family in endothelial–mesenchymal/epithelial–mesenchymal transition (EndMT/EMT) [27].

Observing the role of miRNAs as aortopathy biomarker of aortic dilation and increasing aortic dilation, it has been observed that miR-133a has a special linkage with the aneurysms’ incidence [28]; miR-122, miR-130a, and miR-486 are expressed in BAV; and miR-718 is used to predict aneurysms [29] similar to miR-34a [30]. 

Fibrillin 1 (FBN1) mutations have been found in BAV and aortic dilation. This gene encodes a glycoprotein of extracellular matrix (ECM), which manteins elastic fibers, and is also involved in the linkage of epithelial cells to interstitial matrix. A downregualtion of this gene has been associated with BAV [31]. GATA (sequence for transcription factors for zinc proteins’ binding DNA sequence) variations are involved in BAV: a missense p. Arg202Gln in GATA5 and three synonymous variants—p. Cys274 and p. His302 in GATA4, and p. Asn458 in GATA6 [32]. Alterations in nitric oxide synthase 3 (NOS3) are also associated with BAV. A single nucleotide polymorphism (SNP) is present in aneurysmal and non-aneurysmal BAV [33]. A haplotype within the AXIN-1-protein disulfide isomerase family A member 2 (AXIN1-PDIA2) locus and in the Endoglin (ENG) gene has been found to be linked to BAV [34]. Cilia and excyst have a main role in regulate mitogen-activated protein kinase (MAPK) signaling. An alteration of this mechanism is the cause of an activation of MAPK and the formation of BAV and calcified aortic stenosis [35]. 

## 4. Classification and Nomenclature

Since 1970, several classifications of BAV, derived from pathology, US scan, CT scan, and MR patterns (Table 3), have been proposed [36]. Recently, an international consensus statement developed a classification based on the progression of cusps fusion and geometry of commissurae [37], with particular attention to surgical indications and techniques. 

From this consensus statement, three BAV patterns related to the fusion of cusps and the number of sinuses may be observed. Every pattern should be considered like a schematic-based US short-axis scan at the base of the heart; the ideal circumference of the aortic valve is subdivided into parts like the face of a clock, in which the points over the watch are the coordinates of the anatomical features of the BAV. 

In normal cardiogenesis, endothelium-derived nitric oxide syntethase (eNOS) expression is related to endocardial cells and is dependent upon the shear stress [48,49]. Nitric oxide is the promotor of podokinesis. In this way, cardiac jelly is populated by endocardil cells to make endocardil cushions [50]. In a study on mice, eNOS deficency may cause an alteration of cell migration with impairment in the development of valvular cushions, and an alteration of the function of cardiac neural crest cells has a role in this pathogenetic pattern [7,51].

**Figure 1:**
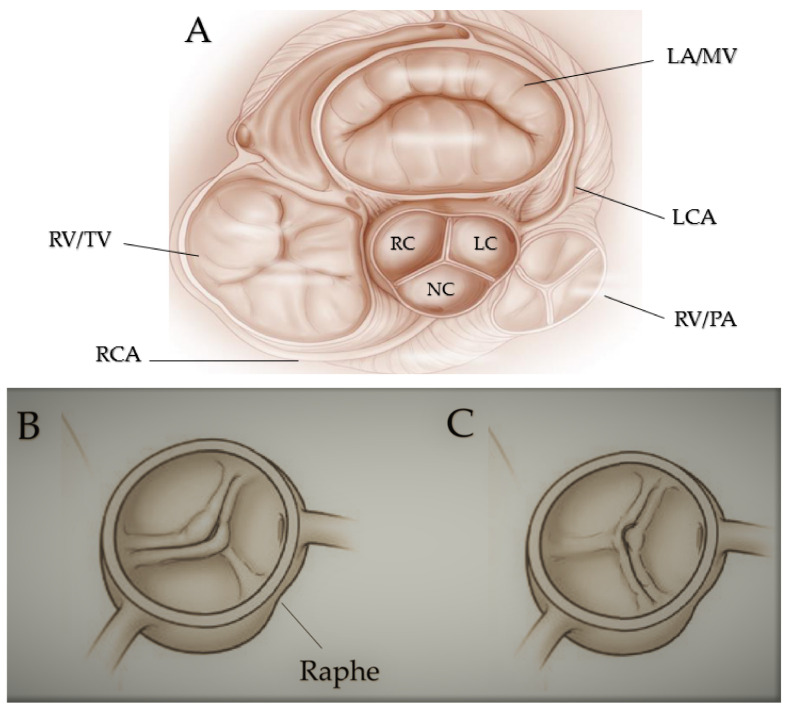
Fused bicuspid aortic valve. (**A**) Represents short-axis normal tricuspidal aortic pattern with anatomical proximities. Cusps’ fusion patterns seen in short heart axis: right-left coronary fusion (**B**), right-non coronary fusion (**C**). All BAVs have three sinuses. Raphe structure is between the fused cusps. Non-fused cusp is prominent in respect to the fused ones. The commissure angle of the non-fused cusp has a degree < 180°. Abbreviations: LA, left atrium; LC, left cusp; LCA, left coronary artery; MV, mitral valve; NC, non-coronary cusp; PA, pulmonary artery; RA, right atrium; RC, right cusp; RCA, right coronary artery; RV, right ventricule; TV, tricuspidalic valve. Licenses Centre Cardiologique du Nord; order date 8 September 2022; order number 5384080341542; publication NEJM; Title: Mitral valve Repair for Mitral valve prolapse.

The first pattern related to embryological events is defined as the fused bicuspid aortic valve (Figure 1) and diagnosed in 90–95% of cases [44] and presents three subtypes defined according to the cusps involved. 

In normal conditions, valve cushions are modelled by an excavation process resulting in fusion of the cusps in case of process alteration [51,52,53,54,55]. It is possible to distinguish three sinuses and the fusion of two of the three cusps. In contrast, the non-fused cusp commissure has an angle of different degrees and generally is more prominent than the fused cusps, as occurs for its sinus compared to the other two sinuses. A fibrous raphe, a predictor of further development of AS [56] between the two fused cusps, has been frequently observed [40,57]. The right–left cusp fusion, observed in 70–80% of patients [58] and often associated with AS and aortic regurgitation (AR) [44], is derived from a mild alteration in the outflow tract septation during embryogenesis and is linked to the formation of aneurysms in every section of the aorta (aortic root, ascending aorta, aortic arch) and frequently characterized by root dilation. An association has also been observed between right–left cusp fusion and aortic coarctation. The right–left cusp fusion is common in genetic syndromes, such as Turner’s one and Shone’s complex and people with Down syndrome [59]. In 20–30% of BAV cases, a proper non-coronary cusp fusion is present, more common among the Asian population [60] and frequently associated with AS in adults [56].

Moreover, it may be observed combined with an alteration of the process involved in the formation of the endocardial cushion, an independent predictor of AR [61]. In children, this phenotype may induce a more rapid development of AS and AR [62,63]. Left non-coronary cusp fusion is only present in 3–6% of patients [37]. 

**Figure 2:**
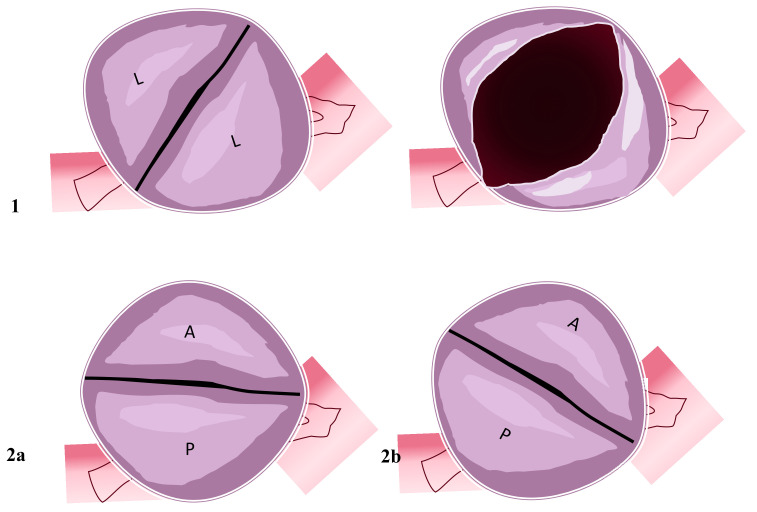
Two-sinus bicuspid aortic valve. Figure represents two cusps’ non-fusion patterns seen in the short heart axis. Aortic valves have two sinuses with two leaflets non-derived from fusion mechanisms. (**1**) Coronary arteries originate from the two sinuses with two lateral leaflets. The opened valve in systole phase has the oval-ball image. (**2a**) In this position, coronary arteries originate from the anterior sinus (right coronary artery) and posterior sinus (common left stem). (**2b**) Right coronary artery and common left stem both originate from anterior sinus. Abbreviations; A, anterior; L, lateral P, posterior.

The second type of BAV is referred to as the two sinuses BAV type (Figure 2).

Its incidence ranges between 5 and 7% of cases [40,44,64]. In this pattern, it is possible to identify two cusps corresponding to homologous sinuses, not depending upon fusion but upon the abnormal embryological constitution. Typically, the cusps are the same in size, a raphe is not present, and the aortic orifice is divided into two portions: laterolateral (Figure 2(1)) and anteroposterior (Figure 2(2a,2b)). In the laterolateral pattern, coronary setup is from each sinus; in the anteroposterior type, coronaries may originate from each sinus or the anterior one. Embryological alteration involved in the laterolateral pattern is secondary to abnormal endocardial cushion formation and positioning. The aetiology of the anteroposterior model is due to abnormal outflow tract septation. The same mechanism in the fused aortic bicuspid valve type is present in this second morphological pattern, but the two-sinus valve may constitute a more severe embryological development alteration [37].

**Figure 3:**
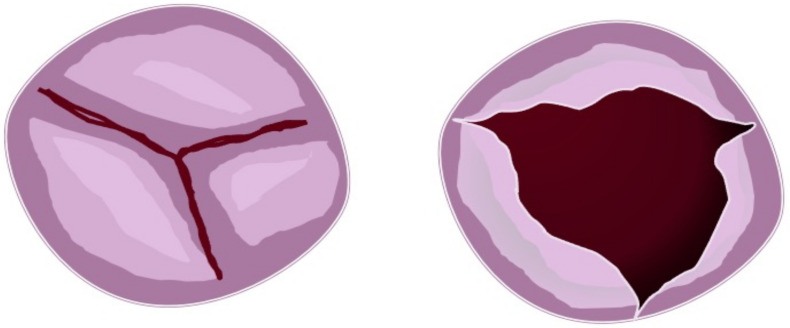
Partial fusion aortic valve. Figure represents three cusps with partial leaflets fusion seen in the short heart axis (**left**). In this case, the opened (**right**) aortic valve is similar to the normal valve but with a more narrow area.

The third BAV type is a partial fusion bicuspid aortic valve (Figure 3), with an unknown prevalence [65]. 

Morphological features are similar to a tricuspid valve with symmetry of the cusps, and the aortic orifice area is less comprehensive than the normal surface. A raphe is localized at the base of each commissure, causing a fixed portion of the cusp to the artic wall. For this reason, this phenotype is also called form fruste aortic valve [66,67,68]. An alteration of normal embryological processes may be identified. Therefore, it is assumed that a mild defect in outflow tract septation and remodelling of aortic valve cushions are present.

The above classifications (Table 3) have implications for daily clinical practice. Siever’s classification is still the most important for diagnosis and surgical indication. The classifications with the determination of the leaflets and the fusion patterns of the commissures are crucial for the development of aortic dilatation [69]. Even aortic valve morphology, flow changes, and prognostic evaluation are well determined by models derived from fusion pattern classification.

## 5. BAV Geometry Types and Surgical Implications

**Figure 4:**
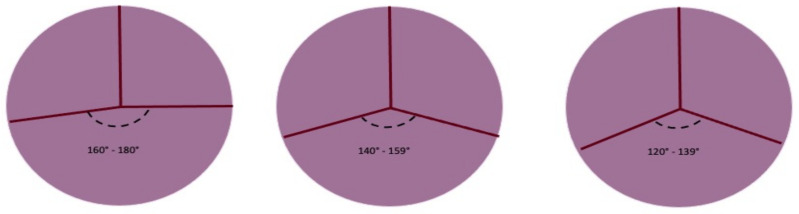
Symmetry of fused bicuspid aortic valve (adapted from Michelena HI et al./European Journal of cardio-thoracic surgery). Figure represents the angles determined by aortic valve leaflets fusion patterns. The length of raphe causes the retraction of fused leaflets and the non-physiological coaptation of the non-fused leaflet with fused leaflets. The geometry of the three patterns can be summarized as symmetrical, asymmetrical, and very asymmetrical The degree of the angle is important for surgical technique.

In every subtype of the previous classification, we can identify the BAV geometrical pattern by evaluating the position of commissures related to the aortic orifice, their angle in the coaptation zone, the presence of raphe, and the morphology and the area of the cusps. In the fused BAV type, it is relevant to establish the relationships between the fused cusps and non-fused cusp and the angle of the commissures of the non-fused cusps (Figure 4). 

When the two fused cusps are retracted over the raphe, AR may develop. Therefore, the coaptation line and angle must be described mainly for surgical indications and practice. The coaptation angle may vary from 180° to less than 150°; to ensure a higher probability of valve repair, the ideal angle should range between 180° and 160°. When the angle approximates 140°, valve sparing and repairing is more complex [70]. Pre-cardiopulmonary bypass transoesophageal echocardiography establishes the coaptation model and commissural angle. 

The two-cusp fusion model, more frequent in AS, has the two cusps/two sinuses feature, and the commissural angle is nearly close to 180° [37]. In partial fusion, the commissural angle resembles the one present in the normal aortic valve. 

Surgical valve-preserving techniques are indicated by the commissural geometry of the aortic valve, as mentioned by a recent paper that also considers the relationship of the aortic root from the virtual basal ring (VBR) to the sinitubular junction [71]. 

## 6. Pathophysiology 

Histological and hemodynamic features have a critical role in understanding BAV development. Histological changes in the aortic wall structure may be ascribed to cystic medial necrosis. The process involved in smooth muscle cells’ regulatory pathways is well known. Extracellular matrix fibrillin 1, abnormally processed by smooth muscle cells, causes the separation of smooth muscle cells from the extracellular matrix layer. After that, MMPs are activated with consequences on the fragmentation of elastin and cellular apoptosis, and the media tunica becomes less prone to flexibility than normal aortic wall [72,73,74].

**Figure 5:**
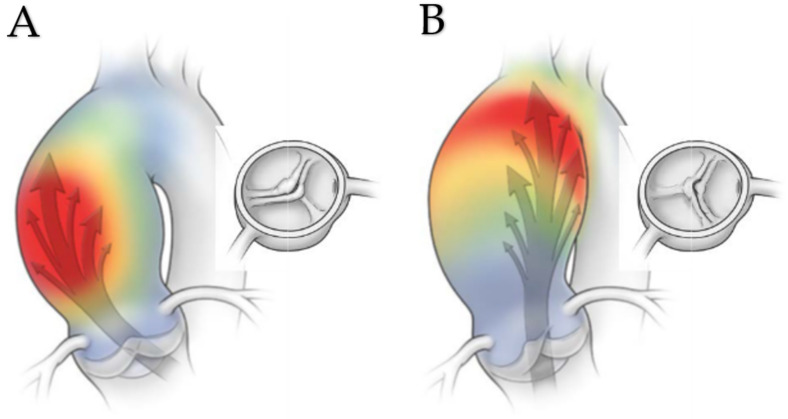
(**A**,**B**). Representation of morphologic chacteristic of the bicuspid aortic valve influencing the pattern of aortopathy. The fusion pattern of the aortic valve cusps is responsible for changes due to shear stress on the aortic wall and in the resulting flow pattern. (**A**) In the right–left fusion model, the jet is directed towards the right anterior wall of the ascending aorta, where it moves in a right-hand helical direction to promote dilation predominantly of the ascending aorta. (**B**) In contrast, in the model characterized by a fusion of the right and non-coronary cusps**,** the jet is directed towards the posterior wall of the aorta, so the model of shear stress of the wall it causes can favor aortic expansion at the internal proximal arch. Licenses Centre Cardiologique du Nord; order date 27 July 2022; order number 5357160571198; publication NEJM; Title: Aortic Dilatation in Patients with Bicuspid Aortic Valve.

Hemodynamic implications cooperate with histological patterns in developing aortopathy in BAV. Biophysics may significantly confirm pathological evidence, given the relationship between hemodynamic and histological patterns. Analysis of the flux, especially in fused bicuspid valves, helps understand the way of aortic dilation, remembering that even a normofunctioning bicuspid valve may cause a flux alteration. A notable contribution to these aspects is due to cineMR of the heart and ascending thoracic aorta [75]: assessing the development of aortic dilation and its complications, such as the dissection, allows for considering flux modification rather than the normal one. For this purpose, the Wall Shear Stress (WSS), peak velocity, normalized flow displacement, and in-plane rotational flow (IPRF) should be observed (Figure 5).

Given that the morphology of BAV influences WSS, this should be evaluated considering its two main axial and circumferential components. Especially in fused patterns and a particular portion of the aorta, WSS may be incidental in aortic dilation (Figure 5). In this case, two distinct models of aortic dilation may be identified: the tubular aortic dilation and the root dilation. The R-L fusion type (Figure 5A) causes more WSS to the root and the outer curvature of the proximal part of the ascending aorta with a lower influence upon the upper tubular portion of the aorta itself; instead, R non-cusp fusion (Figure 5B) exerts a stronger WSS on the convexity of ascending aorta with involvement of the aortic arch [76,77]. Moreover, WSS is also dependent on the degree of AS, given its contribution to a more abnormal flux pattern and the consequent possibility of developing hybrid forms of aortic dilation. 

Root aneurysms may be associated with tubular aortic and arch enlargement. This type of aortic dilation is called root phenotype extended [37,78]. Root phenotype is more frequently associated with aortic dissection, especially in patients who have previously undergone aortic valve replacement (AVR). This is determined by WSS and genetic factors [79]. The ascending phenotype is determined from WSS and the significant curvature of the tubular portion, which can determine a more substantial power of WSS [80,81]. In this context, Sigovan et al. described how the flow jet angle (FJA) and normalized flow displacement (NFD) might act upon the aortic wall, causing dilation [82]. In-plane rotational flow (IPRF), determined in MR imaging, is valuable for measuring rotation flow through a surface. In this sense, the vorticity (ω) and circulation (Γ) are calculated by the integral of vorticity related to a sectional area [83]. Flow volumes are registered as the time integral of forward and backward flow measurements through the aortic surface, thus allowing for the calculation of the systolic flow reversal ratio (SFRR) [84]. Together with biophysical considerations, these parameters are instrumental for a deep CT and RM imaging reading. They may be used during patients’ follow-up, especially in those for whom stratifying the risk of developing and increasing an abnormal aortic diameter is needed. Combining these features with risk factors control and pharmacological approach may be helpful in primary and secondary prophylaxis of aortic dilation.

## 7. Imaging Diagnostic

### 7.1. Echocardiographic Imaging

The role of transesophageal echocardiography (TTE) in diagnosing BAV and its sequelae is well known [38,85,86,87] and mandatory in particular conditions such as AS. It has been estimated that TTE has a sensitivity of 78%, a specificity of 96%, and an accuracy of 93% [38]. In patients with AS, ECG-gated CT is recommended. 

**Figure 6:**
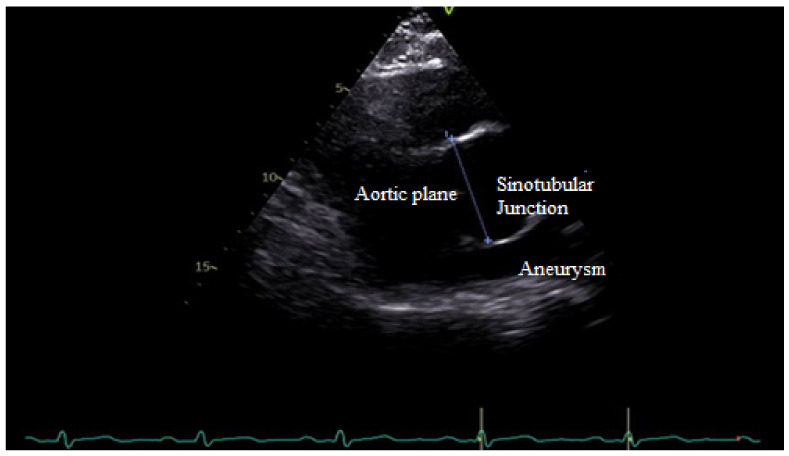
TTE shows enlargement of the sinotubular junction related to R-L cusp fusion.

TTE determines the morphology of the valve, the connected hemorheology, the anatomical features of the root system, the diameter and the wall alteration of ascending aorta, and conditions like the aortic coarctation associated with BAV. In aortic root determination, TTE allows for measuring the sinotubular junction (STJ), especially in some aortopathy related to BAV (Figure 6). 

It is performed using a parasternal short-axis and subcostal short-axis 2D scan, in which the first parameter helps detect two aortic valve cusps. A long axis may be used to investigate the doming in the systolic phase of the fused cusps.

**Figure 7:**
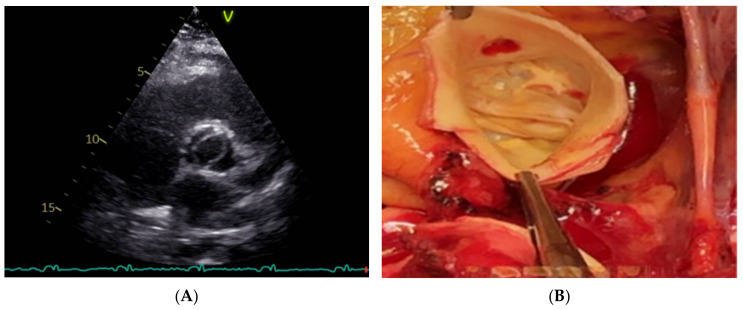
(**A**) TTE. Right non-coronary cusps fusion. (**B**) In the picture from operation theater, it is possible to appreciate the fusion between the right cusp and the non-coronary cusp. Three sinuses are still viewed. Commissural geometrical juxtaposition forms a 180-degree angle.

To describe patterns in BAV classification, a short-axis 2D scan is the best US view because it allows for detecting all the aortic orifices and characterizing cusps fusion and position. It is helpful to determine the presence of raphe and the calcification of the structures related to the components of the aortic valve. Furthermore, TTE, combined with a TC scan, supports determining the relations between cusps, or the angle of commissures, which is important for surgical methods and the width of the sinuses (Figure 7). 

Deeping the role of the US in determining BAV features, the evaluation of the symmetry of the fused cusps related to non-fused cusps is fundamental. Generally, the fused cusps form a new structure that is greater and asymmetrical than the non-fused cusp, while the sinus corresponding to the non-fused cusp is larger than the other two sinuses [88]. 

The valvular function should be well established since flux alterations are present in BAV patterns. Normal functioning valves have to be studied, considering that many of them may evolve into stenosis or regurgitation. Recognizing valves with systolic relevant bending strain, systolic flow models [89], and transaortic fluximetry (peak velocity Doppler) is mandatory. The left ventricular outflow tract (LVOT) is studied with a continuous equation and generally has a larger surface than normal valves. Guidelines recommend employing the peak systolic velocity and mean gradient where the normal ejection fraction is registered [90]. 

In AR, every part of the aortic valve and STJ may be investigated. Prolapse of one or both cusps may be observed, usually associated with dilation of the annulus and root system [91]. TTE may contribute to determining the mechanism of regurgitation and establishing if the valve may be repaired or not; in case of positive indication, TTE supports the identification of repair measurements. To obtain a coaptation zone without residual insufficiency, the commissure angle should reach not less than 160°. It is also relevant to determine the presence of calcification and the mobility of the cusps [86].

Furthermore, through the parasternal long-axis and short-axis images derived by TTE, it is possible to measure the diameter of the ascending aorta segments. In particular, the parasternal long axis may not represent the actual diameter of the aorta correctly [92], but, since aortic diameters are orthogonal to blood flow and X-rays form a 90° angle with vectors of blood flow, the CT scan is valuable in determining every single diameter in aortic segments. Furthermore, TTE registers aortic wall measurements at the end-diastolic phase, taking them from the leading edge to the leading edge. This technique allows for identifying aortic enlargement related to the single patterns of BAV. 

Since there is an agreement of no greater than 2 mm between echocardiography and CT or MR measurements (difference not more than 2 mm), TTE has a primary role during follow-up. It may be performed every 3–5 years if the diameter is normal, every 12 months if the diameter is 40–49 mm, and every 24 months if the stability should be assessed. In the presence of a diameter ranging from 50 to 54 mm, TTE must be repeated every 12 months. The function of the aortic valve should be considered for indication of surgery [93]. 

### 7.2. Cardiac Computed Tomography

**Figure 8:**
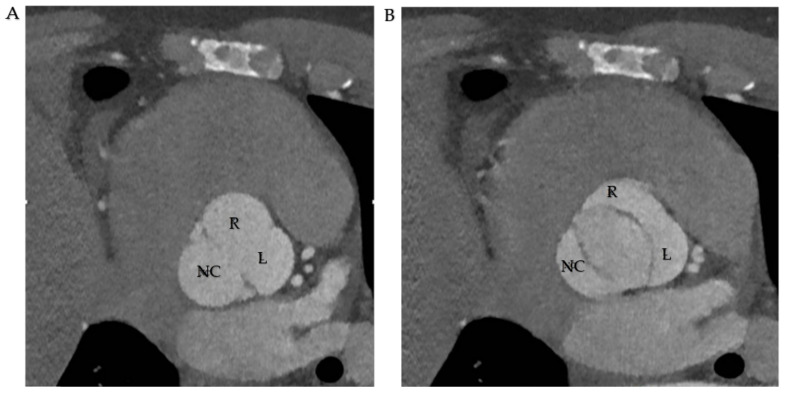
(**A**,**B**). CT scan in R-L cusp fusion. (**A**) Three sinuses are represented. (**B**). Opening mechanism in fusion pattern. Abbreviations; R, right coronary cusp; L, left coronary cusp; NC, non-coronary cusp.

Cardiac computed tomography (CCT) complements US scans in BAV diagnosis. It is relevant in determining aortic dilation, the anatomical edges and correlation with closer structures, and discovering other pathologies correlated with BAV, such as aortic coarctation. Therefore, the radiological protocol is scheduled to determine those features useful for surgeons in the act of choosing traditional surgical approach or transcatheter aortic valve replacement (TAVR) [94] (Figure 8).

In order to diagnose BAV, a 64-slice CT with a venous infusion of 50–100 mL of iodine contrast medium is usually performed. It is helpful to evaluate both systolic and diastolic ECG gating phases, and in the case of BAV, a true commissure or a raphe should be determined [95]. The systolic phase shows the opening pattern of the valve and helps to register the size of the annulus and the leaflets. In the diastolic phase, the edges of the leaflets, their hinge to the aortic wall, the way they close the left ventricle outflow, and the presence of calcifications on their surfaces may be evaluated; coronaries’ imaging should also be evaluated keeping a strict monitoring of heart rate. The role of CCT in determining coronary origin in BAV deserves special mention. Eccentricity of the ostium of the right coronary artery is more frequent (> 20°) than the origin of the left coronary artery. In 95.5% of BAV patients, the obstruction of the right coronary artery is located at the border between the right cusp and the non-coronary cusp. It is also possible to assess the right and left cusps and the right and left coronary midlines. In 97% of BAV patients, the right and left cusps are slightly displaced from the commissure. In 93% of BAV patients, a displacement of less than 20° was noted between the right and left coronary cusps and between the right and left coronary arteries as centered lines [96]. 

Virtual Basal Ring (VBR) software estimates the size and anatomical features [95], especially the anatomical region between the plane passing across the ventricle outflow where this muscular structure encounters leaflets nadir and STJ. The cylindrical geometrical figure can be developed into a rectangular shape, where it is possible to identify hinge regions, sines, and interleaflet triangles. This multiplanar reconstruction (MPR) is particularly significant for surgical technical choices. To have a correct profile of the entire valve, aortic wall measurements should be taken by tracing curved lines in the inner surface of the aorta. 

Every BAV pattern may be localized through a CT scan given the capacity to determine the exact anatomical coordinates as in the echocardiography. The valve orifice may be divided into parts like a clock face, while coronary cusps and non-coronary cusps have the same place as in the TTE. 

Fusion patterns, the presence of raphe, leaflets coaptation, and commissure angles may be identified according to the general classification [37]. 

A recent radiological classification considering a morphological and geometrical approach derived from the valve, commissural orientation, and the aortic annulus shape was developed with the help of a CT scan [97]. The elliptical index of the annulus is measured related to the angle formed by commissure coaptation. Using this approach, three pattern types can be identified. The first type has a low elliptical index (more circular than the others) with a coaptation angle of 160°–180°. The second pattern has a moderate ellipse eccentricity with a coaptation angle estimated between 140° and 159°. Finally, the third type has a very elliptical annulus and a commissural orientation angle of 120°–139°. 

According to the BAV classification, aneurysm phenotypes may be identified on CCT. The RL cusps’ fusion pattern is better linked to root dilation and the initial portion of the tubular thoracic ascending aorta. RN cusp fusion is involved in the dilation of ascending aorta and aortic arch. CCT enables to measure the diameter of the thoracic aorta at different levels, the structure of the aortic wall, the presence of other aortic pathologies, and aortic wall destabilization/intramural hematomas/dissections. The diameter should be measured from the inner wall to the inner wall in the diastolic phase to correctly estimate the magnitude of ascending aorta.

### 7.3. Magnetic Resonance

**Figure 9:**
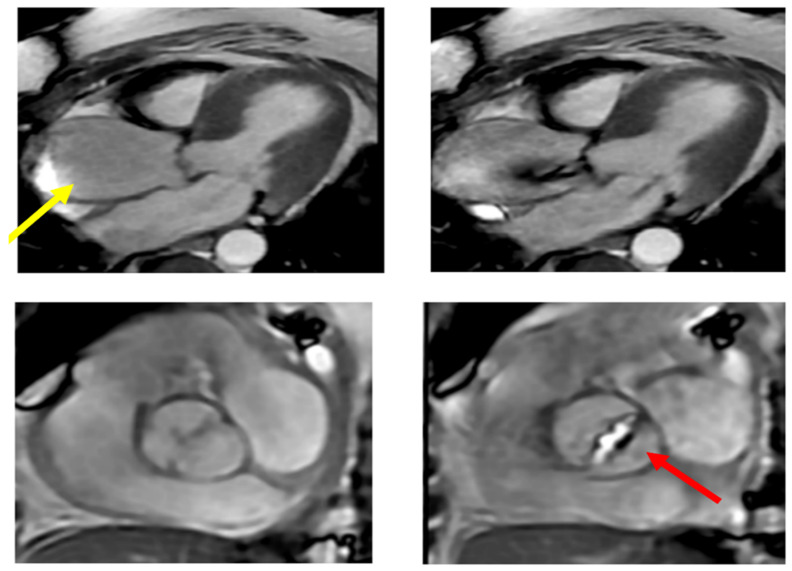
Calcific bicuspid aorta Sievert Type 2 with fusion of the two coronary cusps for a raphe (red arrow). The patient had a transvalvular gradient of 40 mmHg. The ascending thoracic aorta is dilated above the Valsalva sinuses with a maximum diameter of 53 mm measured at the intersection with the right pulmonary artery (yellow arrow).

The contribution of the MR is relevant in those cases in which the echocardiography cannot estimate the morphology of the aortic valve and root and the diameter of ascending aorta and arch (Figure 9). It also has a complementary role in determining the aortic wall structure and the viability of myocardial muscle. It has a main role in determining scarry zones inside a healthy myocardium and the efficiency of cardiac chambers. EF may be estimate with this technique. These features should be matched with other decision elements derived from other imaging techniques to identify the proper surgical indication and forecast the patients’ prognosis. These factors make MR more useful in clinical practice than CT scan regarding functional evaluation [98] (Figure 9).

For hemorheological aspects secondary to BAV, MR is crucial. Time-resolved three-dimensional phase-contrast cardiovascular magnetic resonance (CMR 4D-flow) is necessary for optimal investigation. It allows us to study peak velocity, jet angle, normalized flow displacement, and in-plane rotational flow [75].

Velocity measured through the plane passing along the aortic valve may be associated with its vector figure. Ideally, the angle between the velocity vector and the valve plane is approximately equal to 90°. However, in the presence of BAV, this condition is altered. Therefore, it is necessary to investigate how the velocity vector and the power vector determined by the left ventricular ejection effort influence the blood flux and, consequently, the impact on the aortic wall. This biophysical model considers two particular BAV patterns: The R-L fusion causes a displacement of power against the root portion and to the convex line of the aorta. In contrast, the R-non cusp model shows vector forces directed in the posterior part of the ascending aorta. Interestingly, these power lines are modified in pathological patterns relating to the normal aortic valve, assuming a wider spectrum of action in the CMR 4D flow phase.

MR also contributes to evaluating IPRF and SFRR [75] through a right-handed circular model that describes the geometry of the flux in BAV. IPRF seems higher in R-N-cusp than in the R-L pattern in mid and distal sections of ascending aorta [75,99,100,101,102] and has a higher value even in BAV with the dilated aorta. Higher IPRF values in ascending aortic aneurysm pattern than in the root pattern have also been observed. Rotational flux impacts the circular WSS because it may be possible, in this case, for the conjunction of powers with power vectors effort in double action on the aortic wall. SFRR has higher values in the BAV pattern than healthy persons without no difference between R-L and RN cusp patterns. SFRR levels are higher in ascending thoracic aorta than in the root pattern. In IPRF and SFRR, alterations of effort vectors with alteration of WSS may be observed.

MR imaging helps determine the geometrical and biophysical ascending aorta (AA) features. So far, the morphology of AA is connected to the diameter measure. A retrospective study [103] presented an AA segmentation from the aortic annulus to the emerging of the brachio-cephalic vessel-specific using a 3D segmentation MR software platform to relate the aortic vessel to an idealized cylinder. MR values in every segment were added to reproduce a volume pattern, and the volumetric growth index was determined by comparing baseline and follow-up measurements.

Interestingly results highlighted a difference between diameter measure and volume calculation. In this latter case, the growth index of the aorta was greater than diameter enhancement. Volume representation is more helpful in achieving information from every segment of the aorta than diameter measure, giving a synchronic vision of the idealized cylinder.

AA segmentation by 4D flow MR is a unique technique employed to investigate biophysical aortic features, such as flow rate, distensibility, local strain, and stiffness [104]. Pulse wave velocity (PWV) is determined in aortic regions from Valsalva’s sinuses to the descending aorta (DA). The flow rate is obtained by multiplying the average velocity by the area of a single aortic section. PWV is influenced by diameter expansion, Young’s elastic module, and reduced elasticity (E). PWV decreases when the diameter is larger than a normal aorta diameter and changes when stiffness is greater in pathological patterns than in normal situations.

## 8. Assessment and Treatment

**Figure 10:**
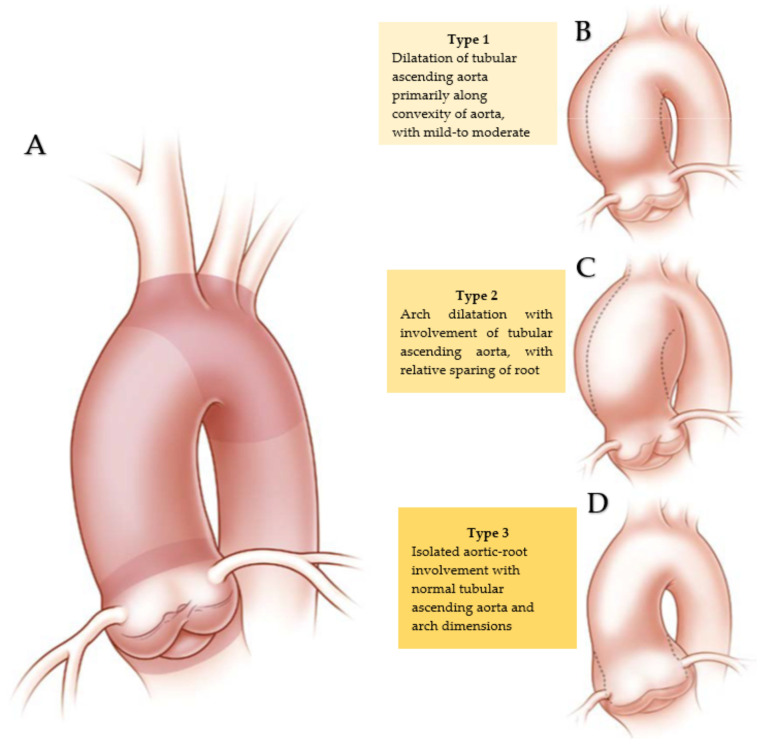
(**A**–**D**)**.** Depict patterns of bicuspid aortopathy revealing the biologic features of the aorta and the three types of bicuspid aortopathy. The three morphological types reported provide a substantial contribution to the best surgical procedure to be used for the treatment of the bicuspid aortopathy. Licenses Centre Cardiologique du Nord; order date 27 July 2022; order number 5357160571198; publication NEJM; title: Aortic Dilatation in Patients with Bicuspid Aortic Valve.

The patterns of aortic involvement guide the surgical choice, and it can be classified into three types (Figure 10). 

Type 1 (B) is the most common type involving dilatation of the tubular ascending aorta with particular regard along its convexity, associated by varying degrees of aortic-root dilatation. Patients who develop this type of morphology have an older age at diagnosis (>50 years). Valvular stenosis and a preferentially RL fusion pattern are disclosed [6,90,91,92]. Type 2 (C) offers as typical feature an isolated involvement of the tubular ascending aorta associated to a relative sparing of the aortic root. Frequently, the morphological type 2 can be extended into the transverse aortic arch, and it has been associated with the presence of the RN fusion pattern [6,63,90,91,92]. Finally, type 3, due to its substantial characteristics, is called the root phenotype, and involves an isolated dilation of the aortic root (D). Its rarity is to be underlined as well as the frequent manifestation in a younger age at diagnosis (<40 years), in the male sex, and the occurrence of aortic regurgitation. Morphological type 3 has been referred to as the form of bicuspid aortopathy that is most likely to be associated with a genetic cause [6,58,64].

**Figure 11:**
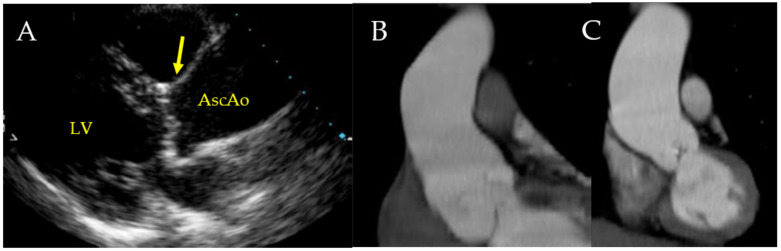
(**A**–**C**). Depicts representative findings on echocardiography and computed tomography (CT). In (**A**), the transthoracic echocardiogram shows normal dimensions of the sinuses of Valsalva (arrow) and a dilated ascending aorta. Ascending aorta denotes proximal ascending aorta, and LV denotes left ventricle. In (**B**,**C**), the CT images reveal dilatation of the aortic root and dilatation of the ascending aorta and proximal arch, respectively. Licenses Centre Cardiologique du Nord; order date 27 July 2022; order number 5357160571198; publication NEJM; Title: Aortic Dilatation in Patients with Bicuspid Aortic Valve.

An early diagnosis of bicuspid aortopathy is likely offered by the use of TTE [42,105,106,107,108]. Although TTE is substantially a method for assessing the morphology of the aortic root and proximal ascending aorta, it is known that the correct visualization of the mid-distal portion of ascending aorta and the arch may present some difficulty in adults. In these cases, both computed tomographic (CT) and MR investigation may be offered a better visualization with a global evaluation of the ascending aorta. In patients who have contraindications to CT or MR, a TTE is suitable for reaching the diagnosis [43,105,109,110]. Likewise, in scheduling serial surveillance, it is more convenient to use MR than CT since it avoids extensive radiation exposure. (Figure 11)

### 8.1. Decision-Making Algorithm for Treatment Option

In patients suffering from bicuspid aortopathy, some risk factors, such as smoking and hypertension, require crucial attention. From a pharmacological point of view, the recent ACC/AHA guidelines recommend using antihypertensive drugs such as beta-adrenergic blockers, angiotensin-converting enzyme inhibitors, and angiotensin-receptor blockers. The use of beta-adrenergic blockers may offer the theoretical advantage of reducing the shear stress phenomenon of the aortic wall, thus avoiding the risk of rupture [111]. Conversely, angiotensin-receptor blockers favor decreasing the aortic growth rate in patients with Marfan syndrome [112]. 

Scheduling a continuous evaluation of the aorta diameter may be indicated in patients with bicuspid aortopathy. If the size of the aortic or ascending root aorta reaches a diameter between 45 and 48 mm, a CT or an MR scan is recommended [43,105,108]. It is important to emphasize that if concomitant indications exist to perform aortic valve correction or associated CABG surgery, a personalized surgical approach is evaluated considering rigorous parameters such as the pattern of aortopathy, the perioperative risk, the skill of the surgeon, and the experience of the referral center [109,113]. In patients in whom the lesion assumes the main characteristic of dilation of the tubular ascending aorta, the various surgical options are directed towards a more or less aggressive approach. The surgeon may choose between isolated supracoronary replacement of the ascending aorta or, in patients with a substantial aortic valve dysfunction associated with aortic root dilation, a replacement of the aortic valve, aortic root, or ascending aorta [114,115,116,117,118,119,120,121,122]. The surgical approach differs substantially in those patients who exhibit bicuspid aortopathy involving dilation of the ascending aorta in association with an aortic arch expansion. The treatment option may be the replacement of the aortic valve combined with the supracoronary replacement of the ascending aorta and with the involvement of the aortic hemiarch. Again, in case of the involvement of this distal part of the aorta, surgical treatment requires more or less deep hypothermia with the circulatory arrest that may be associated with the use of an anterograde or retrograde cerebral perfusion approach [114,115,116,117,118,119,120,121,122].

In patients with isolated aortic root involvement, the surgical option is directed towards the Bentall procedure, which includes aortic valve and aortic root replacement using a mechanical or biological composite valve conduit. A conservative surgical repair revealed excellent results in cases that present an ideal patho-anatomy of bicuspid aortopathy, although patients must be addressed to expert referral centers [120,121,123,124,125]. Indication of the combined aortic valve and ascending aorta replacement surgery should consider nonsurgical factors integrated into the final decision-making process. Therefore, the patient’s lifestyle, the need for long-term anticoagulants, and future reproductive plans in the case of female patients should be considered. A Ross procedure may represent the ideal option for special populations because it uses the pulmonary autograft, which constitutes a living tissue [111,126,127,128,129,130].

Patients with no indication for valve replacement and who reveal dimensions of the aortic root or ascending aorta with a diameter ranging from 45 to 50 mm should be referred for surgery only if they have substantial high-risk characteristics such as a family history of aortic dissection, evidence of sudden rupture, and evidence-based imaging of an aortic growth rate greater than 5 mm per year [115,117,118,119,122,131]. On the other hand, the ratio of aortic area to body height greater than 10 cm^2^ per meter is also effective for patients with short body stature [109,110,132]. If these conditions are insufficient to establish a correct clinical evaluation, an annual reassessment of risk stratification using CT or MR should be reconsidered.

Current ACC/AHA guidelines and the position papers of professional societies recommend a threshold of 5.5 cm and a more individualized approach. COR I and LOE A of ACC/AHA state that in asymptomatic or symptomatic BAV patients with a diameter of the aortic sinuses or ascending aorta higher than 5.5 cm, operative intervention to replace the aortic sinuses, and/or the ascending aorta is recommended. In asymptomatic patients with an aortic root or ascending aorta with a diameter ranging between 5.0 and 5.5 cm and an additional risk factor for dissection (COR 2a, LOE B-NR), surgery is recommended [111,113,133]. 

In other specific clinical conditions, different approaches may be adopted. For asymptomatic BAV patients with low surgical risk and a diameter of the aortic sinuses or ascending ranging from 5.0 to 5.5 cm without additional risk factors for dissection, surgery to replace the aortic sinuses and/or the ascending aorta may be considered if the surgery is performed at a comprehensive valve center (COR 2b, LOE B-NR) [115,117,118,119,120,122,131,133,134]. BAV patients who meet the criteria for replacement of the aortic sinuses may be considered for valve-sparing surgery when the surgery is performed at a comprehensive valve center (COR 2b LOE C-LD) [114]. European guidelines recommend the aortic replacement in patients who experience a diameter of the aortic root or ascending aorta at 5.0 cm or more and when patients have associated risk factors that include coarctation of the aorta, systemic hypertension, family history of dissection, or an increase in the aortic diameter of more than 2 mm per year [93]. International guidelines recommend ascending aortic replacement surgery in patients with a lower threshold (aortic diameter: 45 mm) for whom there is an indication for aortic valve surgery and when valve repair can be performed in an expert center [93,111]. As for patients who received an AVR related to BAV disease and presented with an aortic sinus or ascending aortic diameter greater than 4.0 cm, serial surveillance with lifelong aortic imaging is advisable [135,136].

**Figure 12:**
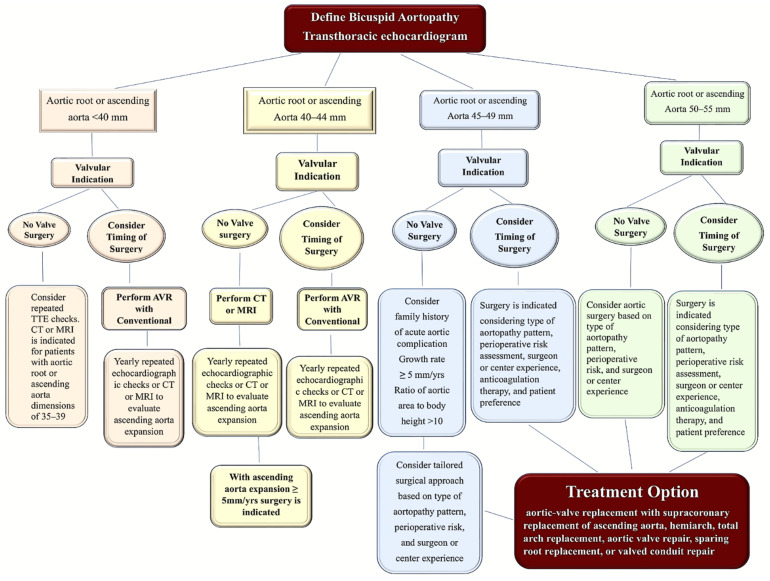
Decision-making algorithm for the management of the bicuspid aortopathy. Abbreviations; AVR, aortic valve replacement; CT, computed tomography; MRI, magnetic resonance imaging; TTE, transthoracic echocardiography.

Finally, the Canadian guidelines recommend the surgical option for an aortic diameter threshold that ranges between 5 and 5.5 cm, also considering the body surface and specific patient risk factors as fundamental criteria, such as the time when the procedure is performed and the nature of the elective aortic replacement [137,138]. Prophylactic surgery is recommended for patients with a lower threshold limit of 50 mm and substantial risk factors for developing an aortic complication, such as rapid aortic growth, concomitant aortic valve disease, and disorders related to connective tissue or genetic syndromes. However, the prophylactic surgery option is not recommended in patients with an increased risk of complications during surgery. Canadian guidelines assume that, since the aortic complications represent a long-term risk that increases with time, they may be prevented if patients undergo elective aortic valve replacement and when aortic surgery is executed in centers with a mortality rate less than 1% [137] (Figure 12).

### 8.2. Special Populations

During pregnancy, women who experience a bicuspid aortic valve with concomitant aortic dilatation may record changes in hemodynamics and the level of the tunica media of the aorta leading to an increased risk of complications. In women who reveal a bicuspid aortic valve associated with an aortic diameter greater than 4.5 cm, general guidelines recommend discontinuing pregnancy. For athletes with aortic root or ascending aortic dilatation greater than 45 mm diameter, regardless of valve dysfunction, guidelines recommend participating in low-intensity events.

For patients who experience symptomatic BAV with severe AS, the transthoracic aortic valve replacement (TAVR) procedure may be considered a valid alternative to AVR after evaluation of patient-specific procedural risks, values, trade-offs, and if executed in a comprehensive valve center (ACC/AHA; COR 2b, B-NR) [139,140,141]. Finally, the familiarity with bicuspid aortic disease, such as that which occurs in the first degree of kinship, should involve marked surveillance for early detection of an asymptomatic bicuspid aortic valve and aortic disease [93,111,142]. 

### 8.3. Surgery in Special Population

**Figure 13:**
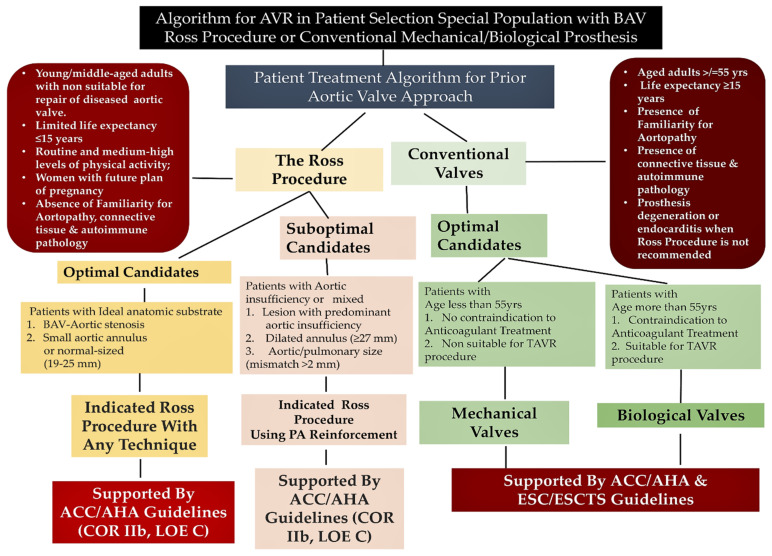
Algorithm for patient special population selection for aortic valve replacement. Ross procedure or conventional mechanical/biological prosthesis may be used according with international guidelines. Abbreviations; ACC, American College of Cardiology; AHA, American Heart Association; BAV, bicuspid aortic valve; COR, class of recommendation; ESCTS, European Society of Cardiothoracic Surgery; ESC, European Society of Cardiology; LOE, level of evidence; TAVR, transthoracic aortic valve replacement.

The Ross procedure with Pulmonary Autograft (PA) is a valuable option for treating bicuspid aortopathy in young or middle-aged patients. PA implanted in an aortic position offers a lasting solution, especially in pregnant women [123,130,143,144,145,146,147,148,149,150,151,152]. Patients who underwent the Ross operation disclosed a retrieval of normal life expectancy, reaching an excellent quality of life with a low number of valve-related complications [127,128,129,130]. The Ross procedure is particularly recommended for women who plan pregnancy because prolonged administration of anticoagulant drugs is not necessary [153,154,155,156]. Consequently, the use of PA as a substitute for the diseased aortic valve has a reduced risk of developing valve thrombosis, thromboembolism, and bleeding compared to the use of mechanical valve prosthesis [157,158]. Furthermore, several studies revealed the superiority of the Ross procedure over other surgical options for AVR in the long term [123,143,144,145,146,147,148,149,150,151,159,160]; nevertheless, ESC/ESCTS does not consider the Ross procedure as a recommendation among surgical options (Class IIb) [93]. Conversely, AHA/ACC guidelines (COR IIb LOE C) recommend using the Ross procedure in patients who require a replacement of the aortic valve [66]. The guidelines support the use of PA in aortic valve and/or aortic root surgery in specific conditions, such as patients no older than 50 years, with non-disabling comorbidity and an aortic stenosis anatomical pattern, and with a small or normal-sized aortic ring. Finally, an experienced surgeon should be involved in the use of pulmonary autograft in young patients with bicuspid aortopathy when AVK anticoagulation is contraindicated or undesirable. We are unaware of any randomized studies comparing the use of Ross operation with cryopreserved aortic homograft for infectious BAV and it is unlikely that such a study will be conducted. Therefore, the current recommendation for the treatment of endocarditis in patients with BAV is based on observational data. Again, evidence from RCTs is lacking for patients who are suitable to receive surgical treatment for a BAV and asymptomatic for a functional or degenerative disorder of mitral valve but who have severe mitral regurgita-tion without a left ventricular dysfunction or dilation, atrial fibrillation, or pulmonary hy-pertension. These patients should undergo early combined mitro-aortic surgery [160,161,162,163,164] (Figure 13).

## 9. Conclusions

BAV remains challenging in everyday clinics. Since patients may present a broad spectrum of anatomy, pathophysiological, clinical, and surgical features, disease classification is complex. A synthetic classification should help elucidate fusion patterns and the geometry of the valve commissures to distinguish valves considered for reparation from valves needing a classical substitution. In the diagnostic field, biophysics may be integrated into regular clinical activity, especially for patients who have no surgical indications but need monitoring to predict the developing enlargement and control risk factors related to dilation velocity.

In the surgical approach, international guidelines focus on the coexistence of the structural pathology and risk factors for aortic dissection and rupture. Therefore, in the new clinical procedures, the alteration of valve structure and aortic enlargement should be considered two aspects of the same disease.

## Figures and Tables

**Table 1:** Narrative review searching strategies.

Items	Specification
Date of Search (specified to date, month and year)	From January 2022 to May 2022
Databases and other sources searched	PubMed
Search terms used (including MeSH and free text search terms and filters)	(BAV OR bicuspid aortopathy OR bicuspid aortic valve) AND (ultrasound OR computed tomography OR magnetic resonance OR US OR CT or MR)
Time frame	Up to May 2022
Inclusion and exclusion criteria (study type, language restrictions, etc.)	English language
Selection process	Two authors independently selected articles after screening for duplicates.

**Table 2:** Gene expression involved in valve and aortic diseases. ACTA 2: alfa actine 2, AXIN: gene encodes a cytoplasmic protein that contains a regulation of G-protein signaling (RGS) domain and a disheveled and axin (DIX) domain, BAV: bicuspid aortic valve, ENG: Endoglin, FBN1: fibrillin 1, GATA (sequence for transcription factors for zinc proteins’ binding DNA sequence), NOS3: nitric oxide synthase 3, NOTCH1 (gene encoding transmembrane proteins), PDIA2: protein disulfide isomerase family A member 2, PECAM-1: platelet endothelial cell adhesion molecule-1, TGF: transforming growth factor, TIMP: tissue inhibitor of matrix metalloproteinases.

Gene Expression	Pathology
miR-146-5p	BAV, aortic aneuurysm (convex region)
miR-21-5p	BAV, aortic aneuurysm (convex region)
miR-17	Aoritc anurysm
miR 21	Aortic aneurysm
miR-34 a	Aortic aneurysm
miR-122	BAV
miR 130 a	BAV
miR-133a	TIMP1,TIMP2, aortic aneurysm
mi-R 143	Aortic aneurysm
mi-R 145	Aortic aneurysm
miR 146-5p	Aortic aneurysm
miR-200	Endothelial-mesenchimal/epithelial mesenchimal
miR-423-5p	BAV, aortic aneurysm
miR-424-3p downregulation	Cell proliferation, apoptosis, endothelial cells alterations, aortic anuerysm
miR-486	BAV
miR-494	PECAM
miR-712	Atherosclerosis, aortic aneurysm
miR-718	Aortic aneurysm
ACTA2	BAV. Aortic aneurysm
AXIN1-PDIA2	BAV
ENG	BAV
FBN 1	BAV
GATA4/GATA5/GATA6	BAV
NOS3	BAV
NOTCH1 (9q34.3)	BAV, outflow tract malformation
TGFb1/TGFb2	Sporadic BAV, Loeys-Dietz syndrome
18q	BAV
5q	BAV
13q	BAV

**Table 3:** BAV classifications (adapted from Michelena HI et al./European Journal of cardio-thoracic surgery). Abbreviations; BAV, bicuspid aortic valve; BAVCon, bicuspid aortic valve consortium; LN, left non-coronary fusion; RL, right–left fusion; RN, right non-coronary fusion.

Author	Nomenclature
Roberts [36] 1970	Anterior–posterior cusps Right–left cusps Presence of raphe
Brandenburg et al. [38] 1983	Clock-face nomenclature: Commissures at 4–10 o’clock with raphe at 2 o’clock (R-L) Commissures at 1–6 o’clock with raphe at 10 o’clock (RN) Commissures at 3–9 o’clock without raphe (L-N)
Angelini et al. [39] 1989	Anterior–posterior cusps Right–left cusps Presence of raphe
Sabet et al. [40] 1999	RL RN LN Presence of raphe
Sievers and Schmidtke [41] 2007	Type 0 (no raphe): anteroposterior or lateral cusps (true BAV) Type 1 (1 raphe): R-L, RN, L-N Type 2 (2 raphes): L-R, RN
Schaefer et al. [42] 2008	Type 1: RL Type 2: RN Type 3: LN Presence of raphe Aorta: Type N: normal shape Type E: sinus effacement Type A: ascending aorta dilatation
Kang et al. [43] 2013	Anteroposterior orientation: type 1: R-L with raphe type; 2: R-L without raphe Right–left orientation: Type 3: RN with raphe Type 4: L-N with raphe Type 5: symmetrical cusps with 1 coronary artery originating from each cusp Aorta: Type 0: normal Type 1: dilated root Type 2: dilated ascending aorta Type 3: diffuse involvement of the ascending aorta and arch
Michelena et al. [44] 2014	BAVCon nomenclature: Type 1: R-L Type 2: RN Type 3: L-N Presence of raphe
Jilaihawi et al. [45] 2016	Tricommissural: functional or acquired bicuspidity of a trileaflet valve Bicommissural with raphe Bicommissural without raphe
Sun et al. [46] 2017	Dichotomous nomenclature: R-L Mixed: (RN or L-N)
Murphy et al. [47] 2017	Clock-face nomenclature: Type 0: partial fusion/eccentric leaflet? Type 1: RN, RL, LN partial fusion/eccentric leaflet? Type 2: RL and RN, RL and LN, RN and LN partial fusion/eccentric leaflet?

## Data Availability

Not applicable.

## Abbreviations

ATA	Ascending thoracic aorta
ACTA	Alfa actine
AS	Aortic stenosis
AR	Aortic regurgitation
AV	Aortic valve
AVK	Anti-vitamin K
AVR	Aortic valve replacement
AXIN	gene encodes a cytoplasmic protein, which contains a regulation of G-protein signaling (RGS) domain and a disheveled and axin (DIX) domain
BAV	Bicuspid aortic valve
CABG	Coronary artery bypass grafting
CCT	Cardiac computed tomography
CineMR	Cine magnetic resonance
CMR 4D-flow	Time-resolved three-dimensional phase-contrast cardiovascular magnetic resonance
COR	Class of recommendation
CT	Computed tomography
DA	Descending aorta
E	Young’s elastic module
EMT	Epithelial–mesenchymal transition
EndMT	Endothelial–mesenchymal transition
ENG	Endoglin
eNOS	Endothelium-derived nitric oxide synthetase
Erb	Tyrosine kinase receptor
FBN 1	Fibrillin 1
FJA	flow jet angle
GATA	sequence for transcription factors for zinc proteins’ binding DNA sequence
IPRF	In-plane rotational flow
LOE	Level of evidence
LVOT	Left ventricular outflow tract
MAPK	Mitogen-activated protein kinase
miRNA	Micro-RNA
MMP	Metalloproteinases
MR	Magnetic resonance
MRI	Magnetic resonance Imaging
NFD	normalized flow displacement
NOTCH1	gene encoding transmembrane proteins
NOS3	nitric oxide synthase 3
PA	pulmonary autograft
PDIA2	Protein disulfide isomerase family A member 2
PECAM	Platelet endothelial cell adhesion molecule
PWV	Pulse wave velocity
SFRR	systolic flow reversal ratio
SMAD 2	similar mothers against decapentaplegic Drosophila gene 2
SNP	single nucleotide polymorphism
STJ	Sinotubular junction
TA	Thoracic aorta
TAVR	Transcatheter aortic valve replacement
TEE	transesophageal echocardiography
TGF	Transforming growth factor
TIMP	Tissue inhibitor of matrix metalloproteinases
TTE	Transthoracic Echocardiography
VSMCs	Vascular smooth muscle cells
VBR	Virtual Basal Ring
WSS	Wall Shear Stress
Γ	circulation
ω	vorticity
